# Long-Term Efficacy of an AI-Based Health Coaching Mobile App in Slowing the Progression of Nondialysis-Dependent Chronic Kidney Disease: Retrospective Cohort Study

**DOI:** 10.2196/54206

**Published:** 2024-11-25

**Authors:** Jianwei Ma, Jiangyuan Wang, Jiapei Ying, Shasha Xie, Qin Su, Tianmeng Zhou, Fuman Han, Jiayan Xu, Siyi Zhu, Chenyi Yuan, Ziyuan Huang, Jingfang Xu, Xuyong Chen, Xueyan Bian

**Affiliations:** 1 Department of Nephrology The First Affiliated Hospital of Ningbo University Ningbo China; 2 Tongji University School of Medicine Shanghai China; 3 Beijing Kidney Health Technology Co., Ltd Beijing China; 4 Health Science Center Ningbo University ningbo China; 5 School of Public Health Health Science Center Ningbo University Ningbo China

**Keywords:** artificial intelligence, chronic kidney disease, eHealth care, mobile app, self-management, kidney function, telemedicine, app, health coaching, CKD, mobile phone

## Abstract

**Background:**

Chronic kidney disease (CKD) is a significant public health concern. Therefore, practical strategies for slowing CKD progression and improving patient outcomes are imperative. There is limited evidence to substantiate the efficacy of mobile app–based nursing systems for decelerating CKD progression.

**Objective:**

This study aimed to evaluate the long-term efficacy of the KidneyOnline intelligent care system in slowing the progression of nondialysis-dependent CKD.

**Methods:**

In this retrospective study, the KidneyOnline app was used for patients with CKD in China who were registered between January 2017 and April 2023. Patients were divided into 2 groups: an intervention group using the app’s nurse-led, patient-oriented management system and a conventional care group that did not use the app. Patients’ uploaded health data were processed via deep learning optical character recognition, and the artificial intelligence (AI) system provided personalized health care plans and interventions. Conversely, the conventional care group received suggestions from nephrologists during regular visits without AI. Monitoring extended for an average duration of 2.1 (SD 1.4) years. The study’s objective is to assess the app’s effectiveness in preserving kidney function. The primary outcome was the estimated glomerular filtration rate slope over the follow-up period, and secondary outcomes included changes in albumin-to-creatinine ratio (ACR) and mean arterial pressure.

**Results:**

A total of 12,297 eligible patients were enrolled for the analysis. Among them, 808 patients were successfully matched using 1:1 propensity score matching, resulting in 404 (50%) patients in the KidneyOnline care system group and another 404 (50%) patients in the conventional care group. The estimated glomerular filtration rate slope in the KidneyOnline care group was significantly lower than that in the conventional care group (odds ratio –1.3, 95% CI –2.4 to –0.1 mL/min/1.73 m^2^ per year vs odds ratio –2.8, 95% CI –3.8 to –1.9 mL/min/1.73 m^2^ per year; *P*=.009). Subgroup analysis revealed that the effect of the KidneyOnline care group was more significant in male patients, patients older than 45 years, and patients with worse baseline kidney function, higher blood pressure, and heavier proteinuria. After 3 and 6 months, the mean arterial pressure in the KidneyOnline care group decreased to 85.6 (SD 9.2) and 83.6 (SD 10.5) mm Hg, respectively, compared to 94.9 (SD 10.6) and 95.2 (SD 11.6) mm Hg in the conventional care group (*P*<.001). The ACR in the KidneyOnline care group showed a more significant reduction after 3 and 6 months (736 vs 980 mg/g and 572 vs 840 mg/g; *P*=.07 and *P*=.03); however, there was no significant difference in ACR between the two groups at the end of the follow-up period (618 vs 639 mg/g; *P*=.90).

**Conclusions:**

The utilization of KidneyOnline, an AI-based, nurse-led, patient-centered care system, may be beneficial in slowing the progression of nondialysis-dependent CKD.

## Introduction

Chronic kidney disease (CKD) is rapidly becoming a widespread noncommunicable chronic disease worldwide, presenting a significant public health challenge. It affects approximately 11%-13% of the global population. It is associated with a high mortality rate, substantial health care costs, particularly in its advanced stages, and a potential need for renal replacement therapy [1-4]. It is projected that by the year 2040, CKD will become the fifth leading cause of years of life lost globally [5]. CKD survivors often grapple with various systemic complications that significantly diminish their quality of life [4,6]. Therefore, mitigating the progression of CKD and managing its complications and comorbidities are critical to improving patient outcomes and reducing the associated risks of end-stage renal disease (ESRD) and mortality [6].

Despite being guided by evidence-based practices, conventional treatment methodologies that rely heavily on multidisciplinary health care teams and patient self-management exhibit limitations in efficiently managing and monitoring CKD [4,7-9]. The complexity of CKD management, given its numerous risk factors, complications, and comorbidities, poses a challenge to clinicians. To address this intricate issue, artificial intelligence (AI) has been introduced as a potential revolution [10]. Recent trends demonstrate that incorporating health information technology into disease management can enhance care management and patient self-care in chronic diseases, such as CKD. Mobile health (mHealth) apps, exemplifying this digital advancement, enable patients to conveniently access health services and information, promoting improved patient engagement and self-management [1,7,11-14]. Although existing studies have demonstrated the advantages of mobile app–based nursing systems in enhancing patient education, self-management, dietary guidance, doctor-patient communication, and disease monitoring, there is relatively limited evidence to substantiate the efficacy of slowing CKD progression of CKD [11,15-19].

In our previous related study, we found that “KidneyOnline,” a patient care system based on a mobile app, has the potential to significantly lower the mean arterial pressure (MAP) and reduce composite kidney outcomes in patients with CKD [7]. However, it is essential to mention that this study had a relatively short follow-up period, and the long-term effects of delayed renal function progression have not been researched. Therefore, the objective of this study was to evaluate the long-term efficacy of KidneyOnline in slowing kidney function decline in patients with CKD compared with conventional care. This study aims to provide robust evidence supporting the use of mHealth apps in CKD management and contribute to the broader acceptance and adoption of eHealth initiatives in chronic disease management.

## Methods

### Population

This retrospective cohort analysis was conducted using KidneyOnline, a mobile app in China. KidneyOnline provides an intelligent patient care system for patients with CKD. Information regarding recruitment for the KidneyOnline program was disseminated through WeChat, allowing many people to download the app and engage actively. The patients signed informed consent forms within the app to confirm their participation. All users from January 2017 to April 2023 were screened based on the recruitment criteria.

Patients were enrolled if they (1) were older than 18 years; (2) met the diagnostic criteria for CKD (ie, an estimated glomerular filtration rate [eGFR] <60 mL/min/1.73 m^2^ or an eGFR less than 90 mL/min/1.73 m^2^, concurrent with albuminuria or hematuria lasting at least 3 months, or as defined by other clinically significant indicators); (3) uploaded at least two distinct data entries upon registration, separated by a minimum of 6 months; (4) were not undergoing dialysis, with a baseline eGFR more significant than 15 mL/min/1.73 m^2^; and (5) were capable and willing to provide informed consent. The exclusion criteria were as follows: (1) inability of the patient to operate a smartphone, (2) intention to initiate dialysis or undergo kidney transplantation within the subsequent 3 months, (3) absence of essential baseline or follow-up data, and (4) suspicion of acute kidney injury. To evaluate the efficacy of KidneyOnline in preserving kidney function, we identified a control group of patients with CKD. These patients underwent renal biopsies at the First Affiliated Hospital of Ningbo University between January 2018 and January 2021 but received conventional care exclusively.

### Intervention

The KidneyOnline intelligent patient care system is a nurse-led, patient-focused, collaborative management system for patients with CKD, complementing standard clinic visits. This system leveraged AI and a health coach team composed of experienced nurses trained by nephrologists, dieticians, and social workers. The system incorporated a smartphone app designed for patients, a web-based clinical dashboard app for health care providers, and a cloud server for efficient data management. KidneyOnline had access to at least 5 integral service elements provided by the system. These included the interpretation of disease conditions and lifestyle interventions, regular checkups, real-time question-and-answer sessions, early warnings, and clinical reminders, as detailed in our previous study [7].

Patients in the conventional care group only received routine face-to-face consultations with doctors in hospitals. They were unable to use the services offered by the KidneyOnline Care System.

### Data Collection

The foundational elements of this system involve structuring patient health data using deep-learning optical character recognition. Generally, patient health data originates from various sources including (1) patient self-reported signs and symptoms, (2) intelligent home devices such as sphygmomanometers, and (3) patient medical history, clinical notes, drug prescriptions, laboratory results, pathology reports, and imaging examinations. Within the KidneyOnline intelligent system, patients upload the data by simply taking photos, after which the intelligent system efficiently extracts the data using deep-learning optical character recognition. Combined with manual verification, an organized database was established, enabling integrated and quantitative analysis of patient health data.

The data of patients in the conventional care group were entered by doctors via the KidneyOnline app, with informed consent from the patients. All electronic data and photographs were uploaded instantly to a secure cloud-based server. All data were encrypted and deidentified in adherence to security and privacy regulations. Only the research staff can access the data stored on the cloud platform.

eGFR results were calculated using the Chronic Kidney Disease Epidemiology Collaboration equation [20]. Blood pressure data were collected via the app, using home-based measurements uploaded by the patients using either a mercury or an electronic sphygmomanometer. Proteinuria measured by the total protein test or dipstick method was converted to albumin-to-creatinine ratio (ACR) using approximation formulas [21].

### Outcomes

The primary outcome of this study was the eGFR slope during the follow-up period. Secondary endpoints included changes in the ACR and changes in MAP.

### Ethical Considerations

This study was approved by the Medical and Research Ethics Committee of The First Affiliated Hospital of Ningbo University (2023110A) and was conducted in accordance with the Declaration of Helsinki. Implied consent was obtained from all participants when they registered on the KidneyOnline app, as the privacy policy included a clause allowing anonymized data to be used for research purposes. All data were anonymized and deidentified before extraction and securely stored in compliance with data protection regulations. No compensation was provided to participants, as this study involved a secondary analysis of existing data. No identifiable images of participants were included in the study or supplementary materials, eliminating the need for additional image consent.

### Statistical Analysis

Data distribution properties are expressed as mean (SD) for continuous variables with a normal distribution or median (IQR) for variables with a skewed distribution. For continuous data, comparisons between groups were performed using the Student 2-tailed *t* test. The chi-square test was used for categorical variables and expressed as percentages.

To account for potential confounding factors between the KidneyOnline intelligent patient care system group and the conventional care group, propensity score matching (PSM) was conducted in a 1:1 ratio without replacement using the optimal method. The matching criteria were based on baseline characteristics including age, sex, BMI, baseline eGFR, baseline MAP, baseline ACR, nephropathy type, and renin-angiotensin-aldosterone system blocker (RASB) or immunosuppressive agent (ISA) treatment.

The eGFR slopes of both groups were estimated using a mixed linear regression model with a random intercept and random slope. This model was adjusted for baseline characteristics including age, sex, baseline MAP, log-transformed ACR, and RASB or ISA treatment. Subgroup analyses were performed after stratification based on sex, median age, median baseline eGFR, median baseline ACR, and median baseline MAP. All the missing data were treated as missing data without imputation. Statistical analyses were performed using the R software (version 4.1.2; R Core Team), and 2-sided *P*<.05 was considered statistically significant.

## Results

### Baseline Characteristics

**Figure 1:**
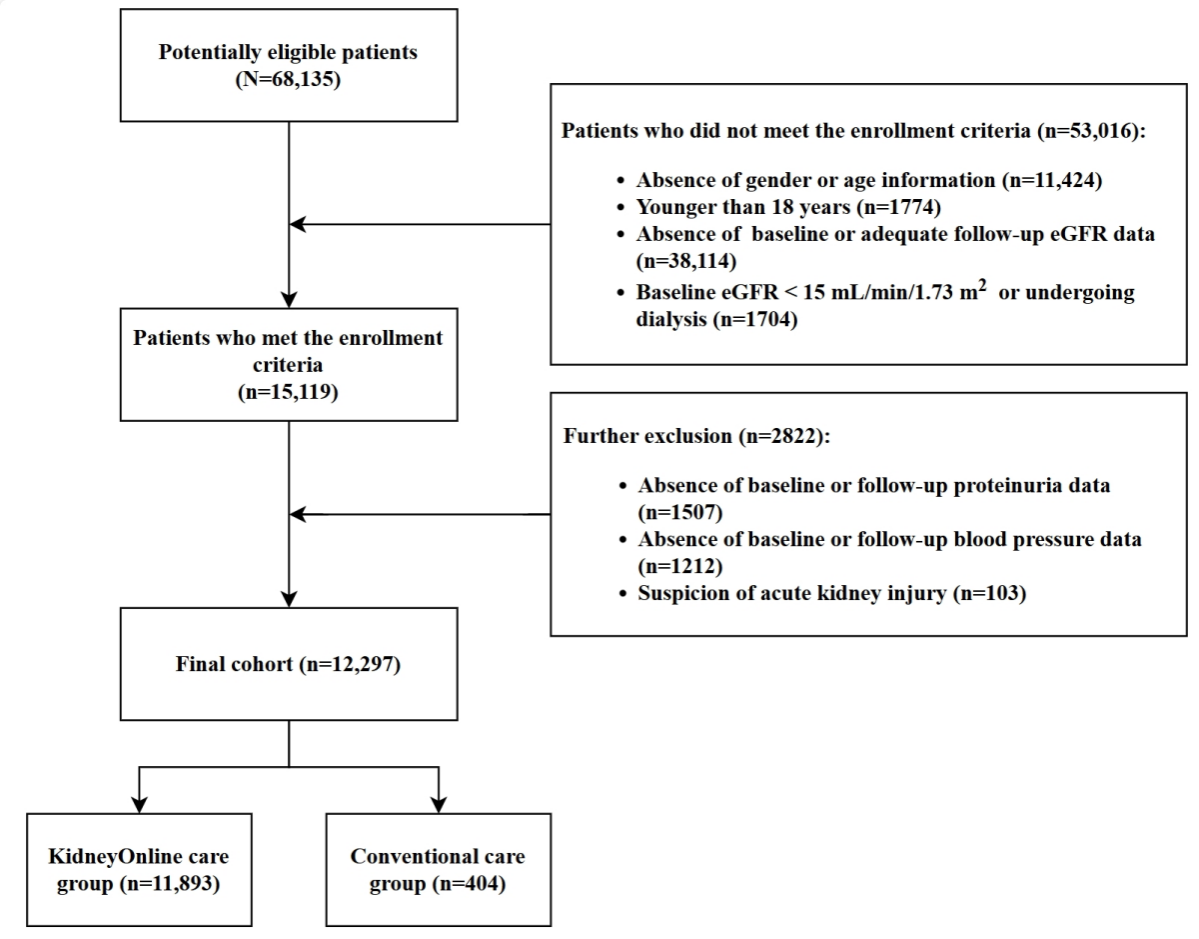
Flow diagram of patient enrollment for the analysis. eGFR: estimated glomerular filtration rate.

Between January 2017 and April 2023, a total of 68,135 potential participants were screened for eligibility. Among them, 12,297 (18%) met the inclusion criteria and were enrolled in this study. Of those enrolled, 11,893 (96.7%) participants were included in the KidneyOnline care group, and 404 (3.3%) participants were included in the conventional care group (Figure 1). The mean age of the enrolled participants was 39.0 (SD 11.0) years, and 6280 (51.1%) participants were female. The mean BMI was 23.1 (SD 5.3) kg/m². In terms of CKD etiology, 5061 (41.2%) patients were diagnosed with biopsy-proven immunoglobulin A (IgA) nephropathy or IgA vasculitis. The baseline parameters revealed a mean eGFR of 84.8 (SD 30.2) mL/min/1.73 m², a median ACR of 410 (IQR 143-1143) mg/g, and a mean MAP of 88.9 (SD 10.7) mm Hg. During the follow-up period, 7152 (58.2%) patients were treated with a RASB, and 3219 (26.2%) patients were treated with ISA (Table 1).

Compared to the conventional care group, patients in the KidneyOnline care group were younger (mean 38.8, SD 10.8 years vs mean 46.0, SD 14.4 years; *P*<.001) and had a slightly lower BMI (mean 23.1, SD 5.4 kg/m^2^ vs mean 23.8, SD 3.6 kg/m^2^; *P*<.001), lighter ACR (median 406, IQR 143-1136 mg/g vs median 551, IQR 162-1562 mg/g; *P*<.001), lower MAP (mean 88.5, SD 10.4 mm Hg vs mean 99.2, SD 13.8 mm Hg; *P*<.001), and lower use rate of ISA (25.9% vs 35.1%; *P*<.001). There were no significant differences in sex, baseline eGFR, or RASB therapy between the 2 groups.

**Table 1:** Baseline characteristics of patients in the total cohort.

		Total (N=12,297)	Conventional care group (n=404)	KidneyOnline care group (n=11,893)	*P* value	
**Age (years)**						<.001
	Mean (SD)	39.0 (11.0)	46.0 (14.4)	38.8 (10.8)		
	Median (IQR)	37.0 (31.0-45.0)	46.0 (34.0-57.0)	36.0 (31.0-45.0)		
**Sex, n (%)**						≥.99
	Female	6280 (51)	206 (51)	6074 (51.1)		
	Male	6017 (48.9)	198 (49)	5819 (48.9)		
**BMI (kg/m^2^)**						<.001
	Mean (SD)	23.1 (5.32)	23.8 (3.55)	23.1 (5.37)		
	Median (IQR)	22.2 (20.1-24.7)	23.5 (21.5-25.8)	22.2 (20.0-24.7)		
**Baseline eGFR^a^** **(mL/min/1.73 m^2^)**						.30
	Mean (SD)	84.8 (30.2)	83.3 (29.8)	84.9 (30.2)		
	Median (IQR)	88.5 (62.1-110.9)	89.5 (60.0-106.4)	88.5 (62.2-111.1)		
**Baseline CKD^b^** **grade, n (%)**						.60
	G1	5970 (48.5)	201 (49.8)	5769 (48.5)		
	G2	3487 (28.4)	102 (25.2)	3385 (28.5)		
	G3a	1286 (10.5)	43 (10.6)	1243 (10.5)		
	G3b	951 (7.7)	34 (8.4)	917 (7.7)		
	G4	603 (4.9)	24 (5.9)	579 (4.9)		
**baseline ACR^c^** **(mg/g)**						<.001
	Mean (SD)	929 (1540)	1270 (1780)	918 (1530)		
	Median (IQR)	410 (143-1143)	551 (162-1562)	406 (143-1136)		
**Baseline proteinuria category, n (%)**						.12
	Normal to mildly increased (ACR<30 mg/g)	1156 (9.4)	32 (7.9)	1124 (9.5)		
	Moderately increased (ACR 30-300 mg/g)	3649 (29.7)	106 (26.2)	3543 (29.8)		
	Severely increased (ACR>300 mg/g)	7492 (60.9)	266 (65.8)	7226 (60.8)		
**Baseline MAP^d^** **(mm Hg)**						<.001
	Mean (SD)	88.9 (10.7)	99.2 (13.8)	88.5 (10.4)		
	Median (IQR)	89.0 (81.7-95.0)	97.7 (90.0-107.1)	88.7 (81.7-94.7)		
**Type of nephropathy, n (%)**						<.001
	IgA^e^ nephropathy or IgA vasculitis	5061 (41.2)	158 (39.1)	4903 (41.2)		
	Membranous nephropathy	1010 (8.2)	77 (19.1)	933 (7.8)		
	Focal segmental glomerular sclerosis	342 (2.8)	16 (4)	326 (2.7)		
	Diabetic nephropathy	108 (0.9)	35 (8.7)	73 (0.6)		
	Hypertensive nephropathy	269 (2.2)	14 (3.5)	255 (2.1)		
	Kidney transplant recipient	54 (0.4)	0 (0)	54 (0.5)		
	Other types of nephritis	5453 (44.3)	104 (25.7)	5349 (45)		
**Drug treatment, n (%)**						
	RASB^f^	7152 (58.2)	230 (56.9)	6922 (58.2)	.65	
	ISA^g^	3219 (26.2)	142 (35.1)	3077 (25.9)	<.001	

^a^eGFR: estimated glomerular filtration rate.

^b^CKD: chronic kidney disease.

^c^ACR: albumin-to-creatinine ratio.

^d^MAP: mean arterial pressure.

^e^IgA: immunoglobulin A.

^f^RASB: renin-angiotensin-aldosterone system blocker.

^g^ISA: immunosuppressive agents.

### Estimation of eGFR Slopes

The average duration of follow-up was 2.1 (SD 1.4) years, and the average number of eGFR follow-ups was 5.4 (SD 3.8). After adjustment for age, sex, baseline MAP, ACR, and RASB or ISA treatment, the mixed linear model showed that the eGFR slope of patients in the KidneyOnline care group was significantly lower than that in the conventional care group (odds ratio [OR] –1.0, 95% CI –2.1 to 0.09 mL/min/1.73 m^2^ per year vs OR –2.7, 95% CI –3.8 to –1.6 mL/min/1.73 m^2^ per year; *P*=.002).

### Baseline Characteristics After PSM

A total of 808 patients (404 per group) were successfully matched using 1:1 PSM. In total, the average age was 46.1 (SD 14.2) years, and 413 (51.1%) were female. The average BMI was 23.8 (SD 4.2) kg/m^2^. The baseline eGFR, MAP, and ACR were 82.7 (SD 30.4) mL/min/1.73 m^2^, 99.3 (SD 14.2) mm Hg, and 582 (IQR 158-1628) mg/g, respectively (Table 2). IgA nephropathy or IgA vasculitis was the most common cause of CKD, accounting for 38.5% (n=311) of cases. A total of 440 (54.5%) patients received RASB and 288 (35.6%) patients received ISA. After 1:1 matching, there were no significant differences in laboratory results or drug treatments between the two groups at baseline (Table 2).

**Table 2:** Baseline characteristics of patients after propensity score matching.

		Total (N=808)	Conventional care group (n=404)	KidneyOnline care group (n=404)		*P* value	
**Age (years)**							.81
	Mean (SD)	46.1 (14.2)	46.0 (14.4)	46.2 (14.1)			
	Median (IQR)	45.0 (35.0-56.0)	46.0 (34.0-57.0)	45.0 (35.0-55.0)			
**Sex, n (%)**							≥.99
	Female	413 (51.1)	206 (51)	207 (51.2)			
	Male	395 (48.9)	198 (49)	197 (48.8)			
**BMI (kg/m^2^)**							.72
	Mean (SD)	23.8 (4.24)	23.8 (3.55)	23.7 (4.84)			
	Median (IQR)	23.2 (21.2-25.7)	23.5 (21.5-25.8)	23.0 (20.8-25.6)			
**Baseline eGFR^a^** **(mL/min/1.73 m^2^)**							.56
	Mean (SD)	82.7 (30.4)	83.3 (29.8)	82.1 (31.0)			
	Median (IQR)	88.1 (59.0-107.3)	89.5 (60.0-106.4)	87.2 (58.1-107.9)			
**Baseline CKD^b^** **grade, n (%)**					.91		
	G1	390 (48.3)	201 (49.8)	189 (46.8)			
	G2	212 (26.2)	102 (25.2)	110 (27.2)			
	G3a	85 (10.5)	43 (10.6)	42 (10.4)			
	G3b	69 (8.5)	34 (8.4)	35 (8.7)			
	G4	52 (6.4)	24 (5.9)	28 (6.9)			
**baseline ACR^c^** **(mg/g)**							.59
	Mean (SD)	1240 (1690)	1270 (1780)	1200 (1600)			
	Median (IQR)	582 (158-1628)	551 (162-1562)	616 (157-1637)			
**Baseline proteinuria category, n (%)**							.54
	Normal to mildly increased (ACR<30 mg/g)	73 (9)	32 (7.9)	41 (10.1)			
	Moderately increased (ACR 30-300 mg/g)	209 (25.9)	106 (26.2)	103 (25.5)			
	Severely increased (ACR>300 mg/g)	526 (65.1)	266 (65.8)	260 (64.4)			
**Baseline MAP^d^** **(mm Hg)**							.87
	Mean (SD)	99.3 (14.2)	99.2 (13.8)	99.4 (14.5)			
	Median (IQR)	97.3 (90.0-106.7)	97.7 (90.0-107.1)	96.7 (90.3-106.6)			
**Type of nephropathy, n (%)**							.83
	IgA^e^ nephropathy or IgA vasculitis	311 (38.5)	158 (39.1)	153 (37.9)			
	Membranous nephropathy	149 (18.4)	77 (19.1)	72 (17.8)			
	Focal segmental glomerular sclerosis	30 (3.7)	16 (4)	14 (3.5)			
	Diabetic nephropathy	66 (8.2)	35 (8.7)	31 (7.7)			
	Hypertensive nephropathy	34 (4.2)	14 (3.5)	20 (5)			
	Other types of nephritis	218 (27)	104 (25.7)	114 (28.2)			
**Drug treatment, n (%)**							
	RASB^f^	440 (54.5)	230 (56.9)	210 (52)		.18	
	ISA^g^	288 (35.6)	142 (35.1)	146 (36.1)		.83	

^a^eGFR: estimated glomerular filtration rate.

^b^CKD: chronic kidney disease.

^c^ACR: albumin-to-creatinine ratio.

^d^MAP: mean arterial pressure.

^e^IgA: immunoglobulin A.

^f^RASB: renin-angiotensin-aldosterone system blocker.

^g^ISA: immunosuppressive agents.

### Estimation of eGFR Slopes After PSM

After 1:1 matching, the average follow-up was 1.7 (SD 1.2) years, and the average number of eGFR follow-ups was 4.6 (SD 2.9). In consistence with the results before matching, the eGFR slope of patients in the KidneyOnline care group was significantly lower than that in the conventional care group (OR –1.3, 95% CI –2.4 to –0.1 mL/min/1.73 m^2^ per year vs OR –2.8, 95% CI –3.8 to –1.9 mL/min/1.73 m^2^ per year; *P*=.009). This suggests that utilization of the KidneyOnline intelligent care system was associated with a delay in the progression to ESRD.

### Subgroup Analysis

To investigate the kidney-protective effect within the KidneyOnline care group across various characteristic populations, we conducted further subgroup analysis. Subgroups were categorized based on sex, median age (<45 vs ≥45 years), median baseline ACR (<719 vs ≥719 mg/g), median baseline MAP (<97 vs ≥ 97 mm Hg), and median baseline eGFR (<86.6 mL/min/1.73 m^2^ vs ≥ 86.6 mL/min/1.73 m^2^). The results indicated that, in all subgroups, the eGFR slope of patients in the KidneyOnline care group was lower than that of patients in the conventional care group. Statistical significance was observed in the subgroups of male patients, patients older than 45 years, patients with worse baseline kidney function (eGFR<86.6 mL/min/1.73 Â m^2^), higher blood pressure (MAP≥97 mm Hg), and heavier proteinuria (ACR ≥719 mg/g; Table 3).

**Table 3:** Subgroup analysis of eGFR^a^ slope.

Characteristic and subgroup		eGFR slope (mL/min/1.73 m^2^ per year)		*P* value
		Conventional care group	KindeyOnline care group	
**Age (years)**				
	<45	–1.4	–0.6	.42
	≥45	–4.2	–1.7	.002
**Sex**				
	Female	–2.7	–1.6	.18
	Male	–3.0	–1.0	.02
**Baseline eGFR (mL/min/1.73 m^2^)**				
	<86.6	–3.0	–0.2	.02
	≥ 86.6	–2.5	–1.5	.18
**Baseline MAP^b^** **(mm Hg)**				
	<97	–2.3	–1.5	.27
	≥97	–3.3	–1.1	.02
**Baseline ACR^c^** **(mg/g)**				
	<719	–1.5	–1.0	.49
	≥719	–4.2	–1.5	.007

^a^eGFR: estimated glomerular filtration rate.

^b^MAP: mean arterial pressure.

^c^ACR: albumin-to-creatinine ratio.

### The Handling of Missing Data

All the missing data were treated as missing data without imputation. As illustrated in Figure 1, among 68,135 patients, 11,424 (16.8%) individuals had missing data on sex and age, while 38,114 (55.9%) individuals lacked baseline or follow-up serum creatinine data for a duration exceeding six months. Additionally, 2719 (4%) patients had missing data on proteinuria and blood pressure follow-up. These patients were excluded from the cohort based on the exclusion criteria. Among the 808 patients who met the inclusion criteria and were successfully matched through PSM, 175 (21.7%) patients had 2 follow-up serum creatinine measurements, while 633 (78.3%) patients had three or more follow-up serum creatinine measurements.

Regarding the follow-up periods, the number of patients with proteinuria data at 3, 6, 12, 18, and 24 months was 473 (58.5%), 419 (51.9%), 374 (46.3%), 332 (41.1%), and 234 (29%), respectively. The number of patients with blood pressure data at 3, 6, 12, 18, and 24 months were 278 (34.4%), 224 (27.7%), 188 (23.2%), 162 (20%), and 122 (15.1%), respectively.

Leffondre et al [22] compared three methods for estimating the slope of eGFR decline over time (linear regression on individual slopes, linear mixed models, and generalized estimating equations). The results indicated that linear mixed models (which are also used in this study) are robust to missing values and irregularly spaced data, where the measurement time points are not fixed. Even with one-third of the data missing, linear mixed models can still accurately estimate the slope of eGFR decline.

### Changes in Blood Pressure, Proteinuria, and eGFR During Follow-Up

**Figure 2:**
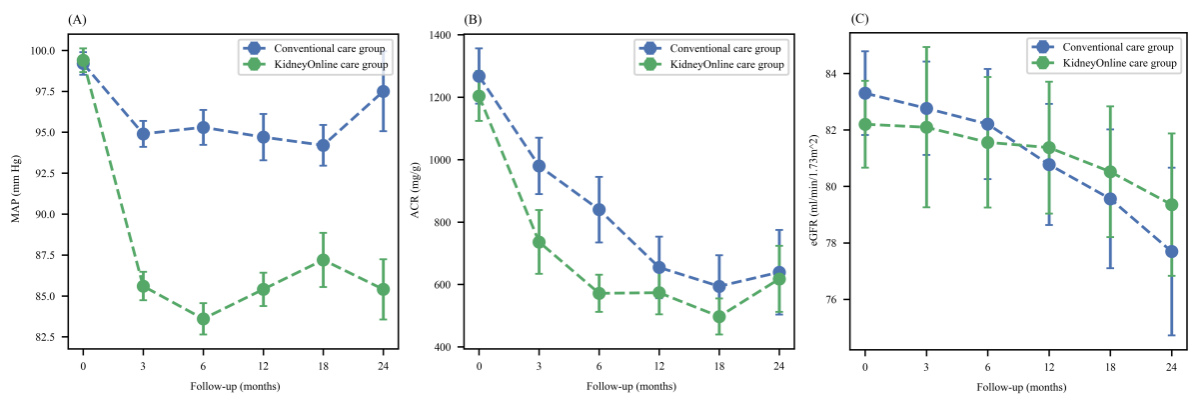
Changes in (A) MAP, (B) ACR, and (C) eGFR during the follow-up period. ACR: albumin-to-creatinine ratio; eGFR: estimated glomerular filtration rate; MAP: mean arterial pressure.

The presence of hypertension increases the risk of a decline in kidney function. Although subgroup analysis revealed that the kidney-protective effect of KidneyOnline care was more pronounced in patients with higher baseline blood pressure, we further analyzed the trend of blood pressure changes in both groups during follow-up. The MAP was similar in both groups (99.4, SD 14.5 mm Hg vs 99.2, SD 13.8 mm Hg; *P*=.87) at baseline. However, after 3 months, the MAP of the patients in the KidneyOnline care group decreased to 85.6 (SD 9.2) mm Hg, while that of the conventional care group was 94.9 (SD 10.6) mm Hg (*P*<.001). After 6 months, the MAP of the KidneyOnline care group further decreased to 83.6 (SD 10.5) mm Hg, while that of the conventional care group was 95.2 (SD 11.6) mm Hg (*P*<.001). During the 24-month follow-up period, the blood pressure level of patients in the KidneyOnline care group was significantly lower than that in the conventional care group and remained stable throughout the follow-up period (Figure 2A).

Albuminuria was indicated by a steep eGFR slope. At baseline, there was no significant difference in the average ACR between the KidneyOnline and conventional care groups (1270 vs 1200 mg/g; *P*=.59). During the follow-up period, the average ACRs in the KidneyOnline care and conventional care groups decreased to 736 and 980 mg/g, respectively, after three months (*P*=.07), and further decreased to 572 and 840 mg/g after 6 months (*P*=.03). However, at the end of 24 months, there was no significant difference in ACR between the 2 groups (618 vs 639 mg/g; *P*=.90; Figure 2B).

The changes in eGFR during the follow-up period are illustrated in Figure 2C. The decline in eGFR in the KidneyOnline care group is more gradual compared to the control group, which is consistent with the eGFR slopes observed in both groups.

## Discussion

### Main Findings

This study describes a nurse-administered smartphone-based patient-centered system designed for disease management. Our results demonstrated that over an average period of up to 2.1 (SD 1.4) years, patients who received the integration of the KidneyOnline intelligent care system, an AI-based mobile app, exhibited a significantly slower decrease of eGFR, lower MAP throughout the follow-up period, and decreased ACR in the early period compared to those received conventional care. This provides a new strategy for long-term improvement of self-management and delayed renal function progression in patients with nondialysis-dependent CKD.

### Analysis of Findings

The findings from this study indicated that using the KidneyOnline intelligent care system may be beneficial for delaying the progression to ESRD in patients with CKD. The eGFR slope is a valuable tool for monitoring kidney function over time, and our data reaffirm its significance. It revealed a substantial contrast between the KidneyOnline and conventional care groups, despite the balanced patient characteristics achieved through PSM. These findings are consistent. Previous research results have evaluated the value of similar telemedicine and intelligent care systems in CKD management [7,8,23]. Recently, in their 3-month prospective study, Li et al [23] found that patients with CKD at stages 1-4 who received dietary and exercise advice, along with motivation through a social media group via a wearable device and smartphone app, exhibited a slower decline in eGFR compared to those receiving conventional care alone (–0.56 vs –4.58 mL/min/1.73 m^2^). Although the results were similar, this study featured a longer follow-up period (averaging 2.1 years). The effect of delaying the decline of eGFR may be attributed to the KidneyOnline care system’s enhancement of the patient’s capacity for self-management, promotion of self-monitoring, encouragement of adherence to lifestyle interventions, and improvement of long-term medication compliance. Remarkably, our subgroup analysis revealed critical information regarding potential beneficiaries of the KidneyOnline care system. We discovered that this system was particularly advantageous for male patients, individuals older than 45 years, and those with worse kidney function, higher blood pressure, and more significant proteinuria at baseline. These findings imply that a personalized approach to CKD care, taking into account patients’ demographics, baseline kidney function, and clinical characteristics, is both feasible and beneficial.

Moreover, it is worth highlighting that those in the KidneyOnline care group displayed more promising trends in critical CKD progression markers. In the initial three months, patients using the KidneyOnline care system showed a significant reduction in blood pressure compared to the conventional care group. This improvement consistently persisted throughout the entire study duration. This reduction in blood pressure could have contributed to the slower decline in kidney function observed in the KidneyOnline care group [24-26]. In parallel, the KidneyOnline care group showed a more pronounced reduction in proteinuria, a potent predictor of CKD progression, highlighting the effectiveness of this intelligent care system [27-30]. Interestingly, by the end of the 24-month follow-up period, there was no significant difference in the ACR between the two groups, suggesting that the ability to sustain long-term effects on ACR through lifestyle and medication adjustments may be limited as CKD progresses, leading to a stabilization of benefits from such interventions. This finding indicates that more intensive or sustained intervention strategies may be required to influence long-term ACR outcomes. Furthermore, the convergence of ACR values also highlights the complexity of managing chronic diseases, underscoring the necessity for continuous innovation and timely adjustment of digital health strategies to meet the evolving needs of CKD patients. Nonetheless, it remains evident that the initial decrease in ACR may have potentially contributed to the kidney-protective effect of the KidneyOnline care system.

### Advantages of KidneyOnline in CKD Management

Even though hundreds of apps for CKD management have been developed, research shows that only a tiny subset of CKD apps received high ratings from patients and nephrologists. This arises because most apps cannot simultaneously fulfill the demands of patient engagement safety, fostering excellent nursing team-patient interaction, providing treatment advice based on evidence-based medicine, and ensuring continuity of care [15,31]. The KidneyOnline intelligent care system is well-equipped to address these demands, offering disease condition interpretation and lifestyle interventions, regular checkups, real-time question-and-answer sessions, early warnings, and clinical reminders.

Many individuals with CKD frequently need to engage in complex disease management strategies. However, they often exhibit limited participation in self-management behaviors, potentially leading to unfavorable outcomes [32]. Therefore, the capacity of an app to enhance patient engagement in self-management is crucial. Singh et al [15] argue that patient engagement in an app should be evaluated from four dimensions: the provision of educational information, reminders or alerts to patients, tracking and summarization of health information, and guidance based on data input by patients. In order to fulfill these requirements, the KidneyOnline system provides lifestyle guidance, including dietary, exercise, and sleep recommendations, using AI to customize personalized meal plans in accordance with the patient’s health status and food preferences. Moreover, it consistently monitors the patient’s health status, blood pressure, dietary choices, and medication adherence through real-time data collection and an integrated intelligent character recognition system. Additionally, the system promptly identifies and alerts to potential risks via its built-in early warning algorithm. These features collectively enhance the patient’s capacity for self-management and safety, thereby enhancing the patient’s prognosis.

Some studies have shown that interactive treatment plans can enhance compliance, safety, and quality of life in patients [33,34]. It is a commonly held belief among both patients with CKD and CKD care providers that mobile apps hold the potential to enhance the self-management of CKD significantly. This enhancement is accomplished by facilitating communication between patients and providers and empowering patients to engage in self-care activities, including adhering to treatment plans. Mobile apps can serve as remote digital platforms for patients and care providers [17]. However, most existing apps designed for CKD management primarily focus on one-sided patient education, failing to incorporate clinical care and health behavior promotion through motivational feedback or interaction with health care providers [13,17,31]. The KidneyOnline care system is equipped to fulfill the functionality mentioned above, enabling health coaches to furnish real-time answers to patient queries promptly. We have also established a renal knowledge graph based on kidney disease: Improving Global Outcomes guidelines for CKD. This feature provides advice based on evidence-based medicine, further augmenting patient care and treatment strategies. Moreover, the KidneyOnline system provides patients with a crucial element of long-term stable management: continuity of care. This is essential for patients’ self-management and their collaboration with health care professionals to decelerate the progression of CKD. However, recent research shows that the continuity of patient-centered care for CKD, as provided by most mHealth apps, falls short of expectations. This deficiency adversely affects patients’ ability to effectively self-manage their condition and hampers the support that health care professionals can offer [31]. A 5-year observational data from the real world have substantiated this notion.

During the COVID-19 pandemic, mHealth apps, such as KidneyOnline, demonstrated significant advantages by providing a secure and accessible platform for chronic disease management. The pandemic introduced unprecedented challenges to health care delivery, including movement restrictions and the risks associated with in-person visits [35,36]. These factors likely shifted patient preferences toward mHealth apps such as KidneyOnline. We observed a substantial increase in the utilization of KidneyOnline during periods of strict lockdown and social distancing. This trend illustrates the pandemic’s role in accelerating the adoption of digital health tools and emphasizes their critical importance in sustaining chronic disease management when access to traditional health care services is constrained. These findings suggest that mHealth tools could be instrumental in maintaining continuity of care and effectively managing chronic conditions during future public health crises. The pandemic has underscored the need to integrate digital health strategies into standard health care practices, ensuring that these tools are readily available and optimized for patient care in routine and crises.

### Strengths and Limitations

The major strength of this study is that KidneyOnline provides a comprehensive approach to CKD management by integrating real-time patient monitoring, AI-driven personalized interventions, and continuous clinical care. This distinguishes it from other CKD management apps, which often focus on singular aspects, like education or symptom tracking, without incorporating interactive features or personalized care plans. This study also offers long-term real-world evidence of KidneyOnline’s efficacy in slowing CKD progression, whereas many studies lack extended follow-up. Additionally, the system’s AI-driven scalability makes it a potential solution for broader health care apps.

However, this study also has some limitations. First, the retrospective nature of the cohort study restricted our ability to establish conclusive causality, which should be further validated through a randomized controlled trial. Second, potential bias may have been introduced by selecting the control group from a different setting. The control group, which was managed solely through conventional care at the First Affiliated Hospital of Ningbo University without any digital health intervention, was compared with patients using the KidneyOnline app. This setting difference may impact comparability, particularly regarding patient engagement, service access, and follow-up care. Although PSM reduced some disparities, it could not entirely eliminate biases. Future studies should consider using more homogeneous control groups or other methods to address these setting-related biases better. Third, we did not analyze other potential endpoints related to the utilization of the KidneyOnline care system, such as the incidence rates of cardiovascular comorbidities, complications, health economic benefits, and hospitalization frequencies. These additional endpoints would have provided a more comprehensive evaluation of our management model for patients with CKD. Fourth, the effectiveness of KidneyOnline may also be influenced by factors such as patients’ levels of technological literacy, motivation to engage in self-management, and adherence to app use, all of which were not measured in this study. It is also important to note that this study population is predominantly comprised of young and middle-aged individuals from China, who generally demonstrate a high level of digital literacy. This demographic characteristic, combined with China’s unique health care and technological environment, might influence the observed effectiveness of the AI-based app KidneyOnline. Moreover, the integration of AI into health care varies widely across different regions, indicating that the outcomes observed in this study might not be universally applicable. Therefore, to fully ascertain the efficacy and applicability of KidneyOnline, future research should include participants from a broader age spectrum, with varied levels of technological literacy, and from different health care systems to enhance the generalizability of the results.

### Conclusions

In conclusion, our research further substantiates the therapeutic efficacy of the mobile app KidneyOnline in delaying the progression of CKD in patients without nondialysis. KidneyOnline is a smartphone-based, nurse-led, patient-oriented management system. Our findings emphasize the potential of AI and machine learning in health care interventions, particularly their capabilities for optimizing management strategies, tailoring personalized treatment plans, and improving long-term medication compliance of patients. Given the increasing global incidence of CKD, there is an urgent need for innovative solutions such as these [37-39]. We encourage further studies to validate these results and to explore the feasibility of implementing digital health interventions on a larger scale.

## Abbreviations

ACR: albumin-to-creatinine ratio
AI: artificial intelligence
CKD: chronic kidney disease
eGFR: estimated glomerular filtration rate
ESRD: end-stage renal disease
IgA: immunoglobulin A
ISA: immunosuppressive agent
MAP: mean arterial pressure
mHealth: mobile health
OR: odds ratio
PSM: propensity score matching
RASB: renin-angiotensin-aldosterone system blocker

## Data Availability

The datasets generated during or analyzed during this study are not publicly available due to business confidentiality reasons but are available from the corresponding author upon reasonable request.
